# Individualized flow-controlled versus conventional pressure-controlled ventilation in on-pump heart surgery (FLOWVENTIN HEARTSURG): study protocol for a randomized controlled trial

**DOI:** 10.1186/s13063-023-07201-7

**Published:** 2023-03-16

**Authors:** Simon Becker, Romina Schnitzler, Martin Rembecki, Johannes Geppert, Christian T. Kurz, Lisa-Marie Wichelhaus, Nina Timmesfeld, Peter K. Zahn

**Affiliations:** 1 Department of Anesthesiology, Intensive Care and Pain Medicine, BG University Hospital Bergmannsheil, Ruhr-University Bochum, Buerkle de La Camp-Platz 1, 44789 Bochum, Germany; 2 Department of Medical Informatics, Biometry & Epidemiology, Ruhr-University Bochum, 44780 Bochum, Germany

**Keywords:** Flow-controlled ventilation, Pressure-controlled ventilation, Cardiac surgery, Cardiopulmonary bypass, Ventilator-induced lung injury, Postoperative pulmonary complications, Electrical impedance tomography, Right ventricular function

## Abstract

**Background:**

In on-pump cardiac surgery, lungs are at high risk of periprocedural organ impairment because of atelectasis formation, ventilator-induced lung injury, and hyperinflammation due to the cardiopulmonary bypass which results in postoperative pulmonary complications in half of this patient population. The new ventilation mode flow-controlled ventilation (FCV) uniquely allows full control of ins- and expiratory airway flows. This approach reduces the mechanical power of invasive ventilation as a possible cause of ventilator-induced lung injury. The scope of FLOWVENTIN HEARTSURG is to compare perioperative individualized FCV with best clinical practice pressure-controlled ventilation (PVC) modes in patients with elective on-pump cardiac surgery procedures. We hypothesize that the postoperative inflammatory response can be reduced by the perioperative application of FCV compared to PCV.

**Methods:**

FLOWVENTIN HEARTSURG is a single-center, randomized, parallel-group trial with two intervention arms: perioperative PCV modes (*n* = 70, PCV group) with an individualized positive end-expiratory pressure (PEEP) and a tidal volume of 6–8 ml/kg predicted bodyweight compared to perioperative FCV (*n* = 70, FCV group) with an individualized PEEP and driving pressure, resulting in a liberal tidal volume. As the primary study endpoint interleukin 8 plasma level is assessed 6 h after cardiopulmonary bypass as a surrogate biomarker of systemic and pulmonary inflammation. As secondary aims clinically relevant patient outcomes are analyzed, e.g., perioperative lung function regarding oxygenation indices, postoperative pulmonary and extra-pulmonary complications, SIRS-free days as well as ICU and total inpatient stays. As additional sub-studies with an exploratory approach perioperative right ventricular function parameters are assessed by echocardiography and perioperative lung aeration by electrical impedance tomography.

**Discussion:**

Current paradigms regarding protective low tidal volume ventilation are consciously left in the FCV intervention group in order to reduce mechanical power as a determinant of ventilator-induced lung injury in this high-risk patient population and procedures. This approach will be compared in a randomized controlled trial with current best clinical practice PCV in FLOWVENTIN HEARTSURG.

**Trial registration:**

German Clinical Trials Register DRKS00018956. Registered on 12 June 2020 (Version 1), last update on 22 August 2022 (Version 4).

**Supplementary Information:**

The online version contains supplementary material available at 10.1186/s13063-023-07201-7.

## Administrative information

**Table:** 

Title {1}	A single center, randomized, parallel group trial to compare perioperative FLOW-controlled VENTilation (FCV) versus conventional pressure-controlled ventilation (PCV) IN adult on-pump HEART SURGery, trial acronym FLOWVENTIN HEARTSURG.
Trial registration {2a and 2b}.	DRKS00018956, German Clinical Trials Register. Registered on 12 June 2020.
Protocol version {3}	23 March 2022, Version 1.5.
Funding {4}	1.) Faculty of Medicine, Ruhr-University Bochum: Annual scholarship as a Clinician Scientist for SB 2.) Ventinova Medical B. V., Eindhoven, the Netherlands: Consumables for flow-controlled ventilation (conventional tube adapter, filter and tubing/cartridge)
Author details {5a}	^1^ Department of Anesthesiology, Intensive Care and Pain Medicine, BG University Hospital Bergmannsheil, Ruhr-University Bochum, Buerkle de la Camp-Platz 1, 44789 Bochum, Germany ^2^ Department of Medical Informatics, Biometry & Epidemiology, Ruhr-University Bochum, 44780 Bochum, Germany
Name and contact information for the trial sponsor {5b}	1.) Research Department, Faculty of Medicine, Ruhr-University Bochum, Contact name: Irmgard Borg, Universitätsstraße 150, 44801 Bochum, Germany, Mail: irmgard.borg@rub.de 2.) Ventinova Medical B.V., Contact name: José van der Hoorn, Meerenakkerplein 7, 5652 BJ Eindhoven, the Netherlands, Mail: jose.van.der.hoorn@ventinova.nl
Role of sponsor {5c}	FLOWVENTIN HEARTSURG is an investigator-initiated and investigator-conducted clinical trial. Thus, the sponsors did neither interfere in study design, collection, management, analysis and interpretation of data nor in the decision to publish any results.

## Introduction

### Background and rationale {6a}

Invasive ventilation can cause ventilator-induced lung injury (VILI) due to excessive airway pressures, tidal volumes (V_t_), and insufficient positive end-expiratory pressures (PEEP) resulting in barotrauma, volutrauma and/or atelectrauma [1]. Additionally, respiratory rate and airway flows turn into focus as new triggers. All determinants of VILI can be summarized in the mechanical power of invasive ventilation resulting in VILI when applied excessively [2]. The underlying pathophysiological mechanism of mechanical power is called dissipated energy which is applied into the airways during inspiration but not fully recovered during subsequent expiration. This energy is “lost “ in the airways thereby causing lung injury [2, 3].

**Fig. 1:**
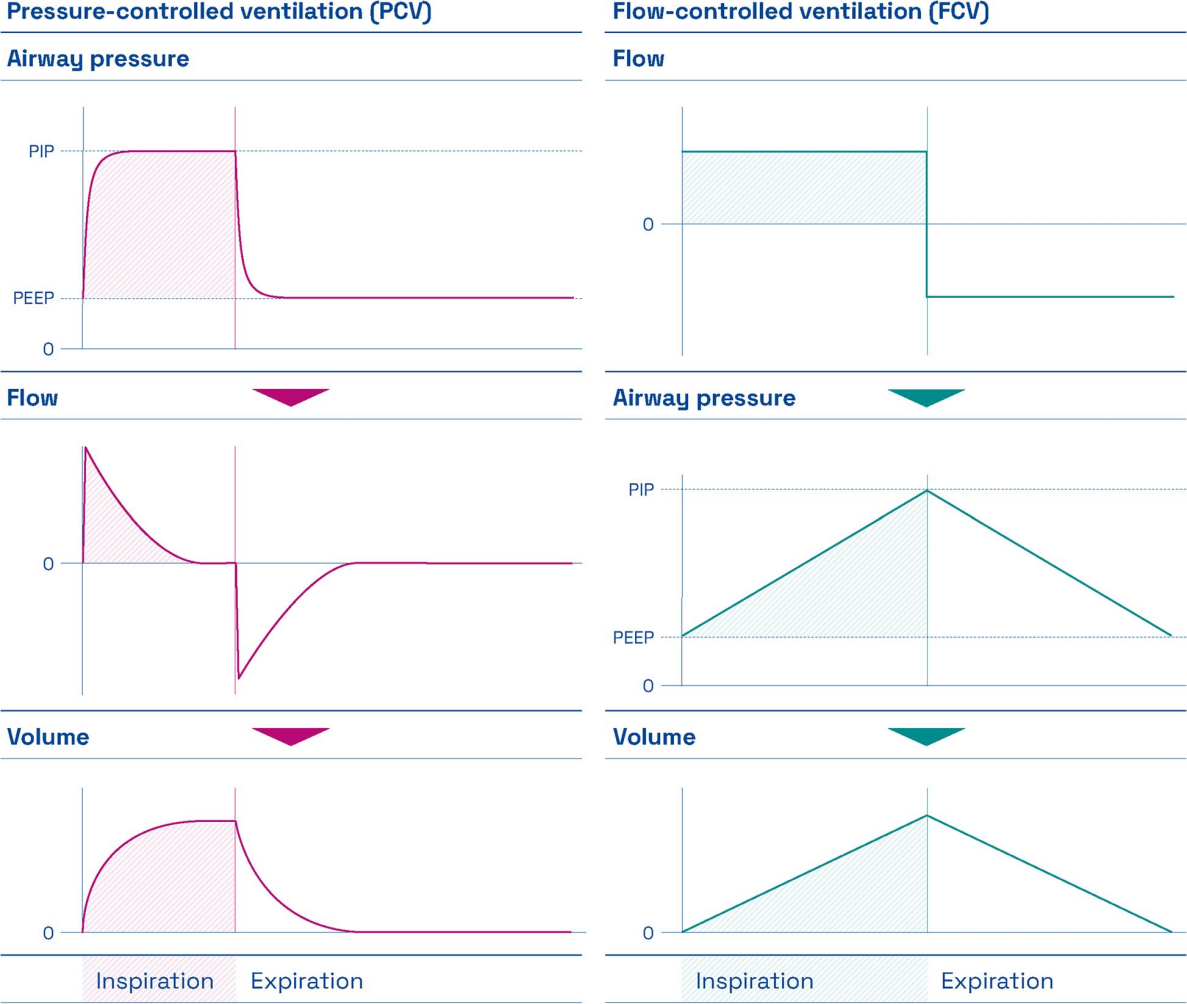
Left column: Course of airway pressures, airway gas flows and ventilated volume during ins- and expiration with conventional PCV (from top to bottom). The relatively abrupt change of airway pressures at the beginning of both ins- and expiration causes a rapid acceleration of airflows into or out of the lungs. The remaining flow profiles during ins- and expiration are characterized by flow deceleration (mid-inspiration and mid-expiration) and eventually end-inspiratory and end-expiratory no-flow phases. Right column: The constant and continuous airway flows during ins- and expiration in FCV (top diagram) effect a linear increase of airway pressures, which are measured in the trachea or at the conventional tube tip when using a conventional tube adapter in FCV (middle). Consequently, ventilated lung volume also increases and decreases linearly during ins- and expiration (bottom)

In conventional mechanical ventilatory modes, inspiration is controlled by pressure (pressure-controlled ventilation, PCV) or volume (volume-controlled ventilation, VCV). In contrast, expiration is driven by the elastic recoil and resistance of the airways/thorax with temporarily high and rapidly changing airway gas flows which cannot be controlled by the operator (Fig. 1). This results in high mechanical power [3].

Since 2017 flow-controlled ventilation (FCV) has been commercially available as a new ventilatory mode (EVONE®, Ventinova Medical B.V., Eindhoven, the Netherlands). With continuous low and constant gas flows during entire ins- and expiration, FCV is the only mode of ventilation in which expiration can be controlled (Fig. 1). This approach reduces mechanical power and dissipated energy of FCV to a minimum in invasive ventilation [3]. In large animal experiments, the application of FCV improved lung function, lung recruitment, homogeneity of ventilation, and reduced lung tissue inflammation in a model of acute respiratory distress syndrome compared to conventional ventilatory modes [4, 5]. Regarding the clinical short-term application of FCV in interventional cross-over studies in lung healthy [6] and obese patients [7], lung function with respect to oxygenation and decarboxylation capacities and regional ventilation distribution were improved compared to conventional volume-controlled ventilation. However, no clinical data exist about the mid-term utilization of FCV in a perioperative context, let alone larger randomized-controlled clinical trials (RCTs). Additionally, even if the theoretical considerations and mathematical models attribute a reduction of mechanical power to FCV [3, 4], clinical data are lacking.

In on-pump cardiac surgery procedures, lungs are not only prone to VILI and periprocedural atelectasis formation. Also, lung ischemia of the alveoli during the cardiopulmonary bypass (CPB) and the consequent lung ischemia/reperfusion injury (LIRI) after CPB termination as well as the extracorporeal circulation itself can cause lung injury which is mediated by hyperinflammation and can manifest at least in temporary organ dysfunction [8].

Every year over one million patients undergo on-pump cardiac surgery procedures worldwide. Due to frequent severe preoperative (pulmonary) comorbidities, invasiveness of the procedures, and the perioperative hyperinflammatory state, cardiac surgery patients are prone to lung injury. Thus, despite fast-track weaning protocols, approximately half of this patient population suffers from some kind of postoperative pulmonary complications (PPCs), ranging from hypoxemia (50%) and pneumonias (below 5%) to the acute respiratory distress syndrome (approximately 1%) [9]. Such postoperative complications extend intensive care unit stays, increase in-hospital mortality, and lead to adverse financial outcomes in health care [10].

As a result, the optimal ventilatory protocol for patients undergoing on-pump cardiac surgery is still under controversial debate. A very recent multicenter RCT (PROVECS trial) could not demonstrate the superiority of a general open-lung concept in various patient-relevant outcomes [9]. Also in non-cardiac surgery, more general and individualized (perioperative) open-lung approaches in large multicenter RCTs (PROVHILO and iPROVE trial) [11, 12] were not convincing in terms of PPCs prevention, even in obese patients (PROBESE trial) [13].

The aim of this trial is to investigate the potential role of individualized FCV as an alternative perioperative ventilation mode in on-pump cardiac surgery procedures and to compare this approach with “best clinical practice” PCV in terms of perioperative inflammation due to VILI, LIRI and invasiveness of the procedure as well as various peri- and postoperative patient-relevant outcomes.

### Objectives {7}

#### Primary objective of the main study

To determine whether perioperative individualized FCV compared to conventional best clinical practice PCV reduces the postoperative inflammatory response due to VILI, LIRI and the cardiac surgery procedure. As the primary endpoint the plasmatic interleukin 8 (IL-8) concentration 6 h after termination of CPB is defined as a surrogate marker of pulmonary and systemic inflammation.

#### Key secondary objectives of the main study

To determine whether FCV compared to PCV benefits the perioperative lung function with respect to oxygenation and decarboxylation capacities, the incidence of PPCs and postoperative extra-pulmonary complications (PEPCs), weaning times from mechanical ventilation and duration of intensive/intermediate care unit stays and time to discharge.

#### Other objectives of exploratory ancillary studies

To determine how an individualized perioperative FCV approach compared to best clinical practice PCV affects

1. the perioperative distribution of air within the thoracic cavity. Data are collected by electrical impedance tomography (EIT) before induction of general anesthesia, during pre- and postoperative mechanical ventilation and postoperative spontaneous breathing. Consequently, different EIT-derived parameters will be analyzed (EIT sub-study, see {12}).
2. perioperative right ventricular function. Functional parameters are assessed by perioperative transthoracic (TTE) or transesophageal (TEE) echocardiography prior to induction of general anesthesia under spontaneous breathing as well as before and after CPB (echocardiography sub-study, see {12}). As mechanical ventilation compromises the pre- and afterload of the right ventricle [14], this question arises when considering the contrary intrathoracic pressure course during FCV compared to PCV (Fig. 1). Additionally, in on-pump cardiac surgery procedures, the right heart is also affected by ischemia–reperfusion injury which impacts on echocardiographic right functional parameters itself [15].

### Trial design {8}

FLOWVENTIN HEARTSURG is designed as a randomized, controlled, single-center, superiority trial with two parallel groups. The group assignment will be performed by block randomization with an 1:1 allocation.

In different ancillary studies, the effect of FCV versus PCV on perioperative lung aeration based on EIT-derived data as well as right ventricular, echocardiographic function parameters will be investigated following an exploratory approach.

## Methods: participants, interventions, and outcomes

### Study setting {9}

Potential study participants are all inpatients of the Department for Cardiothoracic Surgery at the BG University Hospital Bergmannsheil Bochum, Ruhr-University Bochum, Germany.

### Eligibility criteria {10}

#### Inclusion criteria

Eligible study participants are aged 18 years or older with a scheduled elective cardiac surgery procedure including conventional CPB and aortic cross-clamping. As scheduled patients are usually admitted on the day before surgery, a minimum of 12 h between obtaining informed consent and induction of general anesthesia is considered as being appropriate.

#### Patient-related exclusion criteria

- Suspected or confirmed endocarditis and/or pneumonia
- Preoperative (oral) immunosuppressive medication
- Participation in another perioperative interventional trial
- The disability to consent independently to study participation.

#### Procedure-related exclusion criteria

- Emergency or salvage procedures like in type A aortic dissection or acute myocardial infarction
- Patients scheduled for beating heart coronary artery bypass grafting excluding complete aortic cross-clamping, ventricular assist device implantation and heart transplantation.

### Who will take informed consent? {26a}

On the working day before the surgical procedure, the study information will be handed out in paper form to potential candidates after assessing in- and exclusion criteria. The patient is revisited not earlier than after 30 min in order to provide sufficient time to go through the information. Depending on the patient’s specific needs, physicians who are part of the Trial Management Committee (TMC) will explain the study context face-to-face as well as the interventions and perioperative care of each study participant, e.g., additional examinations and the collection of blood samples. Furthermore, the study participants’ rights are communicated, in particular the possibility to terminate study participation at any time without any adverse medical treatment except of the intervention. When all remaining questions have been clarified, the patient can either consent to or deny study participation.

### Additional consent provisions for collection and use of participant data and biological specimens {26b}

Not applicable as the written consent does contain the provisions for the collection and the use of participant data as well as blood samples within the main and ancillary studies specified in the original study protocol.

For future research including analysis of plasma probes, a new votum from a Research Ethics Committee (REC) and additional participant consent must be obtained (see {33}).

## Interventions

### Explanation for the choice of comparators {6b}

Perioperative PCV is a commonly applied mode of invasive ventilation and is preferred to volume-controlled ventilation for some reasons like better patient comfort and lower work of ventilation [16]. Especially for postoperative ventilatory care and ICU patients in need of invasive ventilation, PCV modes like “biphasic positive airway pressure” (BIPAP, Draeger Medical GmbH, Lübeck, Germany) are commonly utilized. Therefore, perioperative PCV with an individual compliance-based best PEEP [12] and driving pressures (DP) ensuring a lung-protective V_t_ of 6–8 ml/kg predicted body weight (PBW) [17] has been chosen for the control group as currently known best clinical practice.

In contrast, perioperative FCV with a compliance-based best PEEP and DP having positive effects on lung function and aeration in pre-clinical mid-term application [4] is utilized in the intervention group.

### Intervention description {11a}

#### General setting

General anesthesia is induced with sufentanil (bolus of 0.3–0.5 μg/kg), propofol (initial bolus of 0.5–1.5 mg/kg following titration depending on the Bispectral index), and pancuronium (bolus of 0.05–0.1 mg/kg) i.v. according to departmental standards. As no volatile anesthetics can be administered technically by the FCV ventilator, total intravenous anesthesia is maintained in both groups by continuous sufentanil (0.5–0.6 μg/kg/h) and propofol infusion (4–6 mg/kg/h) for an intraoperative Bispectral index of 40–50 during the pre- and intraoperative period. During CPB, propofol infusion is suspended and sevoflurane is administered systemically through the membrane oxygenator of the extracorporeal circulation.

After intubation with a conventional tube (ID 8–8.5 mm) and verification of correct tube position by capnometry and auscultation in both groups, patients in the FCV group are additionally equipped with a conventional tube adapter (Ventinova Medical, Eindhoven, the Netherlands). Group-specific invasive ventilation is initiated in a supine position at a PEEP of 5 cm H_2_O and a DP for a V_t_ of 6–8 ml/kg PBW in both groups. PBW is calculated according to PBW = 45.5 + 0.91*(height in cm − 152.4) for female and PBW = 50 + 0.91*(height in cm − 152.4) for male participants.

The respiratory rate in the PCV and airway flows in the FCV group are set to maintain sufficient decarboxylation (arterial pCO_2_ 35–45 mmHg) throughout perioperative mandatory ventilation. Fraction of inspired oxygen (FiO_2_) of the respective respirator is kept in both groups at 0.8 by default throughout the pre- and intraoperative phase.

#### Initial PEEP trial in both groups

**Fig. 2:**
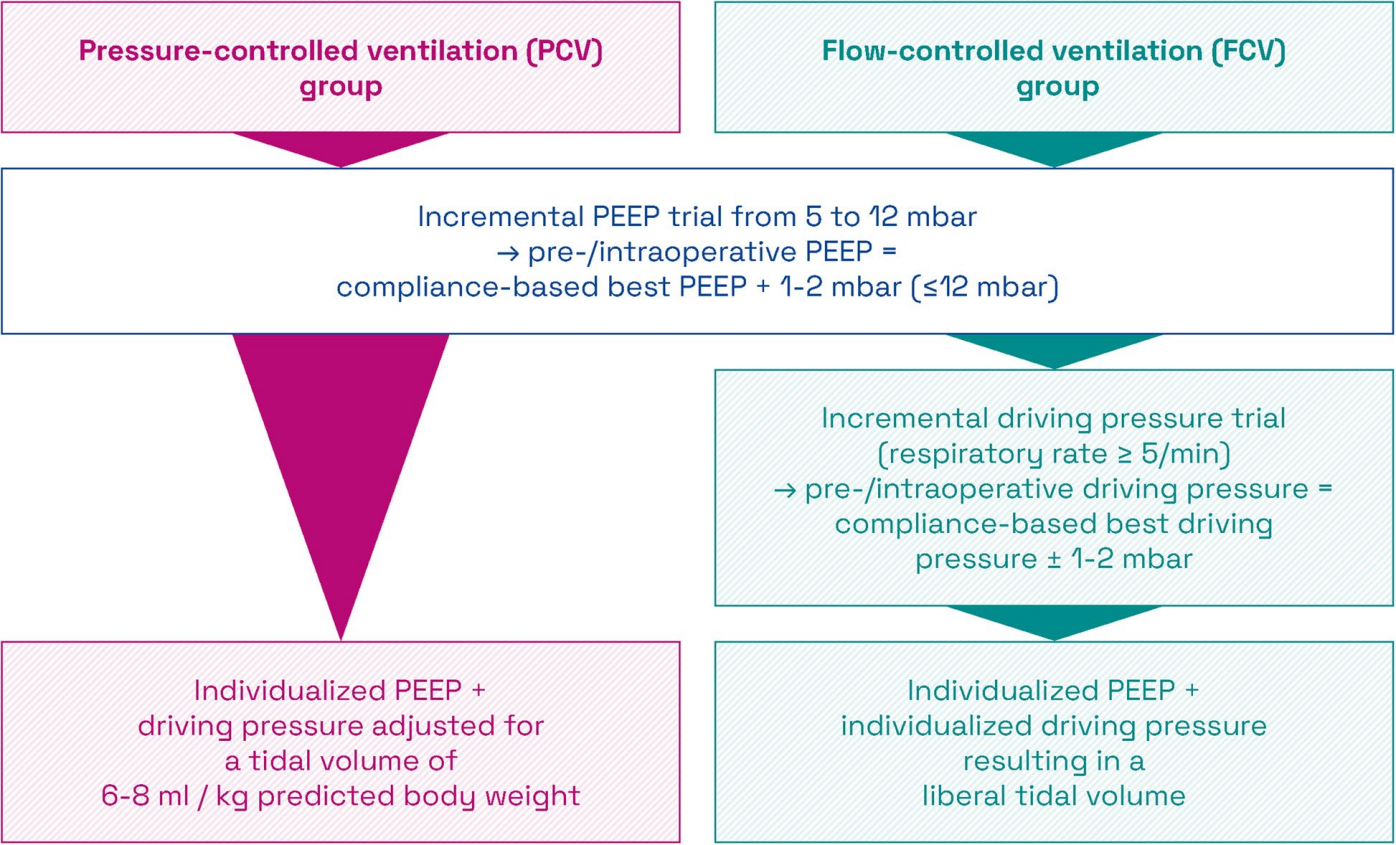
Brief overview of interventions in the PCV and FCV group with respect to pre- and intraoperative respirator settings. PEEP, positive end-expiratory pressure

In both groups, an incremental PEEP trial follows from 5 to 12 cm H_2_O in 1 cm H_2_O steps with a constant DP which ensures V_t_ of 6–8 ml/kg PBW at the very first PEEP level. As the PEEP trial proceeds, dynamic compliances (C_dyn_) of the respiratory system are calculated [18] and the best PEEP level is defined as the one with the highest C_dyn_ [4, 19]. If patients indicate no hemodynamic instability, for example with the need for passive leg raising, lungs in both groups are ventilated with the compliance-based best PEEP + 1–2 cm H_2_O but ≤ 12 cm H_2_O in order to reduce lung de-recruitment and atelectrauma throughout the pre- and intraoperative phase (Fig. 2) [4, 12, 19]. In the PCV group, the DP is adjusted to maintain a V_t_ of 6–8 ml/kg PBW during the pre-, intra- and postoperative phases until weaning from invasive ventilation (Fig. 2).

#### Secondary driving pressure (DP) trial in the FCV group

In the FCV group, an additional incremental DP trial in 1 cm H_2_O steps is initiated after the PEEP trial. This trial evaluates if the V_t_ increases over-proportional to the C_dyn_ on the previous DP level when the DP is increased by 1 cm H_2_O. As soon as the increase of V_t_ is just proportional, lower, and/or a respiratory rate of 5/min is reached, the DP trial is terminated. In the FCV group patients are ventilated with a DP consistent with the highest C_dyn_ ± 1–2 cm H_2_O during the pre- and intraoperative period [4] (Fig. 2). This approach favors a liberal V_t_ and focuses on a reduction of the respiratory rate and airway flows in order to minimize mechanical power [4].

#### Postoperative respiratory settings

After postoperative admission on the ICU under invasive ventilation, another incremental PEEP trial is conducted in the PCV and PEEP + DP trial in the FCV group in order to individualize group-specific ventilation according to the postoperative condition and the upright patient position of 30–45°. In the ICU, PCV is delivered through the BIPAP modus for patient safety and comfort by default. FiO_2_ is reduced to not less than 0.4 depending on the individual oxygenation capacities.

Patient weaning of invasive ventilation is initiated 3–4 h after CPB end in the case of no new postoperative hemodynamic instability, excessive drainage loss nor gross new pathologies according to the postoperative chest x-ray with the need for intervention. For initiation of weaning continuous propofol infusion is stopped and piritramide (0.1–0.15 mg/kg actual body weight) is administered i.v. If respiratory weaning cannot be commenced due to the above-mentioned criteria, group-specific ventilation will be maintained for up to 24 h after CPB. If the participant still fails extubation by that time, ventilatory settings will be at the discretion of the intensivist in charge and not controlled by study personnel any longer.

PEEP and DP trials, as well as pre- and intraoperative interventions, are all administered and conducted by the same anesthesiologist being in charge of protocol adherence together with a specially trained senior cardiac anesthesiologist.

### Criteria for discontinuing or modifying allocated interventions {11b}

Both in the PCV and FCV group, PEEP levels are allowed to be reduced in 1 cm H_2_O steps and ventilation to be temporarily suspended during internal mammary artery preparation or at any other critical time point depending on the surgeon`s needs in analogy to the PROVECS trial [20]. If the sole reduction of PEEP is not sufficient in the FCV group, the DP might as well be cut down in 1 cm H_2_O steps to an acceptable surgical level. However, PEEP must not fall below 5 cm H_2_O and V_t_ not below 6 ml/kg PBW during the perioperative phase in both groups. The standard application of airway recruitment maneuvers is not intended due to limited clinical benefit and a potentially injurious effect [9]. However, recruitment is mandatory in all procedures with a full aortotomy for deairing the left heart chambers before declamping the ascending aorta. Additionally, recruitment might be unavoidable in case of low oxygenation capacities, especially after CPB termination. Therefore, standardized recruitment maneuvers can be performed at the discretion of the anesthesiologist in charge up to a peak airway pressure of 30 cm H_2_O for a maximum length of 10 s and can be repeated depending on the individual patient needs.

### Strategies to improve adherence to interventions {11c}

The application of ventilatory interventions in both study groups is performed by the same anesthesiologist who is only in charge of protocol adherence. Additionally, all respiratory settings are recorded and documented electronically in an anesthetic report intraoperatively or Patient Data Management System after ICU admission which both can be accessed retrospectively in order to ensure study protocol adherence.

### Relevant concomitant care permitted or prohibited during the trial {11d}

If a participant requires cardiopulmonary resuscitation during the group-specific ventilation, it is mandatory that the ventilation mode is changed to volume-controlled in order to assure a sufficient V_t_. The violation of the designated perioperative ventilation protocol due to this incident or any other operational disturbances for more than 5 min will be reported as an adverse event and is additionally defined as a study stop criterion. As further study stop criteria, we determined more than one cardiopulmonary bypass time and intraoperative death solely due to major surgical complications.

### Provisions for post-trial care {30}

Participants enrolled in the study are covered by the business liability insurance of the BG University Hospital Bergmannsheil for negligent harm during the perioperative period including study-related procedures like blood collection. This will cover the additional health care and compensations for damages by claims pursued through the courts.

All respirators in the study are CE-marked and dispose about additional insurance that covers direct harm in association with the correct use of these medical devices.

### Outcomes {12}

#### Primary outcome of the main study

As the primary outcome mean final values of IL-8 plasma concentration 6 h after CPB termination will be assayed by enzyme-linked immunosorbent assay (ELISA).

Previous studies in cardiac surgery could demonstrate lower postoperative plasmatic IL-8 concentrations after protective low tidal volume ventilation and/or higher PEEP level administration [21, 22]. In a sub-study of the PROVHILO trial in non-cardiac surgery, postoperative plasmatic IL-8 concentrations were higher in the group that developed PPCs [23]. Moreover, IL-8 is one of the key cytokines in LIRI [8, 24] and recent data suggest IL-8 levels to be associated with an increased risk of 28-day mortality in patients with acute respiratory distress syndrome in a panel of 6 biomarkers [25]. In this first large clinical trial of individualized FCV, IL-8 plasma concentrations function as a surrogate biomarker for the specific objective and hypothesis of the proposed project.

#### Important secondary outcomes of the main study

1. Oxygenation indices at different perioperative time points (Table 1, “art BGA”)
2. Plasma IL-8 concentrations at different perioperative time points (Table 1)
3. Perioperative ventilation pressures (mean and peak airway pressure, driving pressure), C_dyn_, airway resistances, and mechanical power of ventilation [26] up to 24 h after termination of CPB
4. Weaning times from mechanical ventilation
5. Incidence of postoperative pneumonias with the need of antibiotic treatment during the complete inpatient stay (suspected and/or confirmed, see below)
6. Incidence of postoperative pulmonary complications (PPCs) during the first seven postoperative days including compound outcome analysis and single PPC analysis. PPCs encompass (modified from [20])
  - New invasive ventilation
  - Non-invasive ventilation
  - Post-extubation respiratory acidosis (pH ≤ 7.3 and PaCO2 > 45 mmHg)
  - Reintubation
  - Readmission to ICU due to pulmonary reasons
  - Mild hypoxemia defined as an PaO2/FiO2 < 300 but ≥ 200
  - Moderate hypoxemia defined as an PaO2/FiO2 < 200 but ≥ 100
  - Severe hypoxemia defined as an PaO2/FiO2 < 100
  - Acute respiratory distress syndrome (ARDS) according to the Berlin criteria [27]
  - Implantation of a veno-venous ECMO
  - Suspected pneumonia defined as new pulmonary infiltrates plus 2 or more of the following clinical parameters: temperature > 38.5 or < 35.5°C, leucocyte count > 12 or < 4/nl and purulent secretions including antibiotic treatment
  - Confirmed pneumonia defined as pulmonary infiltrates and microbiological germ proof of tracheal aspirations
  - Bronchoscopy
  - Pleural drainage due to pleural effusions
  - Pleural drainage due to pneumothorax
7. Incidence of postoperative extra-pulmonary complications (PEPCs) during the first seven postoperative days including compound outcome and single PEPCs analysis. As PEPCs the following items are defined
  - Sepsis and septic shock (as defined in [28])
  - Postoperative coronary intervention
  - Postoperative wound infection with the need for antibiotic treatment
  - Other sites of infection with the need for antibiotic treatment
  - Pericardial tamponade or other bleeding with intervention such as re-sternotomy
  - Implantation of any device for extracorporeal life support (ECMO, Impella) or intra-aortic balloon pump
  - Non-pulmonary ICU readmission
  - Acute kidney injury with a Kidney Disease: Improving Global Outcomes (KDIGO) stage 2 or 3 [29]
  - Postoperative delirium diagnosed by the Confusion Assessment Method for the ICU (CAM-ICU) [30]
8. Systemic inflammatory response syndrome (SIRS) (according to [28]) free days during the first seven postoperative days
9. Lengths of intensive/intermediate care unit stays and time to discharge
10. Hospital readmission and mortality rate after one and 6 months post-surgery (Table 1, phone surveys)

Regarding the more patient-relevant secondary endpoints of FLOWVENTIN HEARTSURG with respect to both PPCs and PEPCs, these were validated as primary (composite) endpoints in large multicenter ventilation trials of the past decade [9, 11–13, 17].

#### Outcome parameters for ancillary studies

1. Sub-study on cardiovascular-ventilation interactions (n≥40/group)
  - Perioperative echocardiographic evaluation of pulmonary circulation and right ventricular function parameters at three different times, (1) preoperative TTE before induction of anesthesia (baseline), (2) TEE 1 before CPB, and (3) TEE 2 30 to 60 min after CPB (Table 1). Parameters include [31]
    i Pulmonary acceleration time and estimated pulmonary artery pressures during ins- and expiration
    ii Right ventricular index of myocardial performance (RIMP)
    iii Tricuspid annulus velocity S’
    iv Tricuspid annular plane systolic excursion (TAPSE)
    v Right ventricular outflow tract fractional shortening (RVOT FS)
    vi Fractional area change (FAC)
    vii Longitudinal strain analyses by speckle tracking echocardiography.
    For explorative analyses, final values of each right function parameter at the defined times as well as the relative changes from baseline to intraoperative data and relative changes between pre- and post-CPB measurements will be assessed.
  - Serum concentrations of CK-MB and high-sensitivity troponin up to postoperative day 5 (Table 1)
  - Plasmatic brain natriuretic peptide (BNP) concentrations and relative changes from baseline (i.e. before induction of general anesthesia) up to postoperative day 5 (Table 1)
  - Central venous oxygen saturations at 8 perioperative time points up to 24 h after CPB (venous BGA, Table 1)
  - Perioperative catecholamine concentrations up to 24 h after CPB (Table 1)
2. Sub-study on perioperative lung aeration parameters based on electrical impedance tomography (EIT) data (n≥50/group)
  Parameters include [32]
  i Global inhomogeneity index
  ii Center of ventilation
  iii Regional ventilation delay
  iv Surface of ventilated area
  v Dorsal fraction of ventilated area [33].

EIT recordings are performed at 6 perioperative times: (1) patient breathing spontaneously in supine position before induction of general anesthesia (baseline), (2) after individualization of preoperative, group-specific ventilation, (3) after postoperative ICU admission and re-individualization of group-specific ventilation, (4) on pressure support ventilation with a PEEP of 5–6 cm H_2_O and support pressure of 6 cm H_2_O during weaning from mechanical ventilation, (5) 30–60 min after extubation, (6) 24 h after CPB end (Table 1). The explorative analyses will include final values of each parameter as well as relative changes from baseline, i.e., the spontaneously breathing patient preoperatively.

**Table 1:**
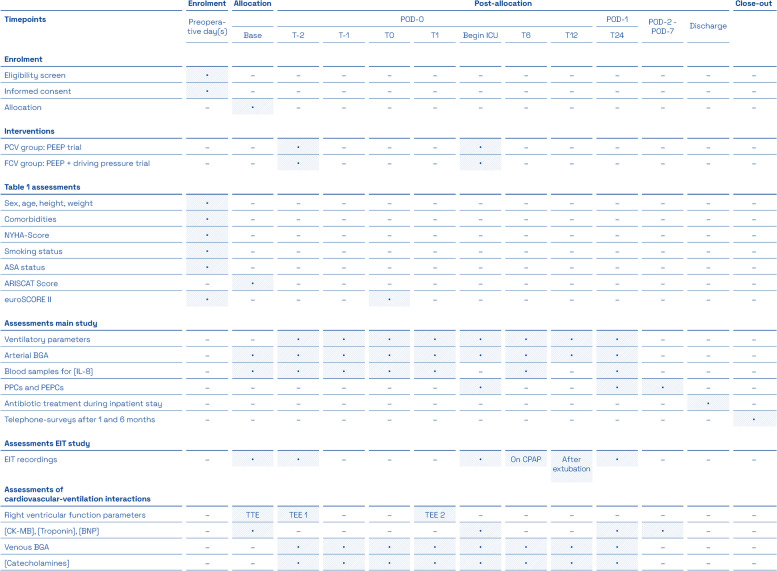
Time schedule for enrolment, allocation, interventions and assessments in the main and ancillary studies. Calculations for the Assess Respiratory Risk in Surgical Patients in Catalonia (ARISCAT) Score [34] can only be completed when SpO_2_ is measured at the time of patient arrival in the induction room. The EuroSCORE II [35] is recalculated after CPB based on the actual intraoperative cardiac surgical interventions. Additional arterial BGAs are conducted simultaneously to the EIT recordings on CPAP and pressure support ventilation during weaning as well as 30–60 min after extubation. Close-out of participants is after the second phone survey 6 months after the cardiac surgery procedure. The first phone survey is conducted one month postoperatively. Post-allocation time points: Base, patient breathing spontaneously before induction of anesthesia; T−2, after group-specific individualization of ventilation and before skin incision; T−1, 15 min after thoracotomy; T0, after CPB termination and transfusion stop from the extracorporeal circulation; T1–24, appropriate hour after CPB end. BGA, blood gas analysis; BNP, brain natriuretic peptide; CK-MB, creatine kinase MB; CPAP, continuous positive airway pressure ventilation during weaning; PEEP, positive end-expiratory pressure; PEPCs, postoperative extra-pulmonary complications; POD, postoperative day; PPCs, postoperative pulmonary complications

### Participant timeline {13}

The participant timeline is shown in Table 1.

### Sample size {14}

The sample size was calculated according to the study hypothesis. Based on the previous results of protective ventilation protocols in the setting of on-pump cardiac surgery [21, 22], the effect size of the intervention on postoperative plasmatic IL-8 concentrations was estimated as moderate being well aware about the exploratory nature of this study of mid-term clinical FCV utilization. Under these assumptions (Cohen’s d = 0.5), the total sample size required to achieve a power of 1-β = 0.8 for a two-sample *t*-test at a two-sided level α = 0.05 resulted in 2*64 = 128 [36]. As the primary endpoint is obtained 6 h after termination of CPB, most dropouts will be due to a second cardiopulmonary bypass phase within this time frame or other in {11d} defined stop criteria in estimated 10% or less of the cases. Therefore, an additional 10% of patients will be randomized summing up to a total sample size of 128*1.1 = 140.8 ≈ 140 randomized study participants.

### Recruitment {15}

As most patients are accompanied for 12–14 h during the perioperative period by the same anesthesiologist for protocol adherence only one participant per day is generally feasible. Depending on this availability, preoperative patients will be screened for eligibility criteria on the day before surgery.

In order to motivate for trial participation, all suitable patients are informed about the possibly improved perioperative medical care, not only by the intervention but especially during the postoperative period when being supervised by an extra medical doctor in the ICU. However, medical treatment except of interventions is at the discretion of the respective anesthesiologist or intensive care physician in charge. Generally, recruitment is performed in an informative way with plenty of time to answer all questions and encounter individual concerns. Additionally, every patient is made aware of the option to cancel the study participation at any time without any effect on further medical treatment except of interventions.

## Assignment of interventions: allocation

### Sequence generation {16a}

Randomization to the control (PCV) or intervention (FCV) group is performed by a computer-generated schedule with an 1:1 allocation using permuted blocks of lengths 4 and 6 in order to ensure sufficient concealment.

### Concealment mechanism {16b}

The online-based software REDCap® will only release the randomization code to group-specific intervention assignments after entering sex and age as well as the affirmation of inclusion and negating exclusion criteria of a recruited patient. Assignment is only performed if the participant`s time slot for the procedure is confirmed by the respective OR manager.

### Implementation {16c}

The allocation sequence was generated by independent staff from the Department of Medical Informatics, Biometry & Epidemiology, Ruhr-University Bochum. Patient recruitment and enrolment is organized and executed by trained personnel from the Department of Anesthesiology, Intensive Care and Pain Medicine, BG University Hospital Bergmannsheil. Finally, interventions are assigned only by the principal investigator (SB) using the online-based software REDCap® with no access to the allocation sequence.

## Assignment of interventions: blinding

### Who will be blinded {17a}

Due to application of interventions with different respirators and ventilatory equipment, care providers and assessors cannot be blinded during group-specific mechanical ventilation in FLOWVENTIN HEARTSURG. However, trial participants are blinded at any time point until individual trial close-out after 6 months post-surgery.

As soon as the patients are extubated, blinding of at least study assessors is possible. At the earliest, this can be achieved 6 h after CPB as most patients are in the process of weaning or already extubated. At the latest from 24 h after CPB onwards, all involved study assessors are masked as well as data analysts at a later time. Blinding of these parties is not lifted before a fixed data set of each outcome parameter is created.

### Procedure for unblinding if needed {17b}

Not applicable as per default care providers and assessors are not blinded until the intervention withdrawal—which is when patients are eventually weaned and extubated during the postoperative care or after 24 h of group-specific ventilation post CPB.

### Plans for assessment and collection of outcomes {18a}

#### Primary and secondary outcomes of the main study

For the analysis of plasmatic IL-8 concentrations 6 h after CPB (primary study endpoint), central venous blood will be collected under aseptic conditions from a central venous catheter in 4.0 ml EDTA tubes. Consequent laboratory processing is initiated within 50 min after collection and includes centrifugation of full blood at 2000 g for 10 min and freezing of 200 μl aliquots at − 80 °C. All assessors have been specifically trained for this basic laboratory work. One aliquot per participant will be analyzed in duplicate by an ultrasensitive IL-8 Human ELISA Kit, according to the manufacturer’s instructions (Thermo Fisher Scientific, Darmstadt, Germany). ELISA plates will be occupied with aliquots starting with the most recent and ending with the very first patient in order to diminish inter-assay variability. ELISA assessments are conducted only by one single specialized laboratory staff member unknown to the group assignment in order to further decrease inter-observer variability and to promote the reliability of the ELISA results.

For the collection of main secondary outcomes like oxygenation indices, ventilatory parameters, PPCs and PEPCs, participants are accompanied continuously from in- to extubation and are visited on postoperative days 1, 3, 5, and 7. All assessors are physicians with a special training for maximum inter-rater reliability.

#### Ancillary studies

1. *Perioperative cardiovascular-ventilation interactions:* The assessors of right ventricular function parameters by preoperative TTE and intraoperative TEE are senior anesthesiologists in cardiac anesthesia with a special diploma in these diagnostic procedures. Assessments are conducted at distinctly defined times ({12} and Table 1).
2. *Perioperative lung aeration parameters based on EIT data:* EIT raw data are recorded on six serial perioperative times ({12} and Table 1) with a patient-matching EIT-belt positioned between the 4^th^ and 6^th^ intercostal space. EIT parameters will be analyzed by a trained and blinded assessor with the matlab tool EITdiag and PC-version of PulmoVista® (Draeger Medical GmbH, Lübeck, Germany).

A trial-specific case report form (CRF) for the collection of data for the main and ancillary studies can be found in additional file 1.

### Plans to promote participant retention and complete follow-up {18b}

Patients are allowed to withdraw study participation at any time and for any reason. However, as interventions are only applied when the patient is under general anesthesia and most study data are collected within the first 24 h after CPB while the patient is being either under general anesthesia or shortly after awakening from an open chest cardiac surgery procedure, withdrawal rate taking together with the dropout rate (see {14}) and missing data is thought to be less than 10% of the cases during the whole study period.

In order to reduce the risk of loss to follow-up during phone surveys up to 6 months post-surgery, at least one phone number of a close relative is recorded at the time of recruitment.

### Data management {19}

After inclusion, every patient receives an individual paper CRF, which is filled out as the study proceeds individually. These CRFs are stored at a central place up to postoperative day 8 for assessors who visit patients up to postoperative day 7. From then onwards, CRFs are stored in numerical order in a secure place with restricted access. Every quartile of the total sample size, which is after 35, 70, 105, and 140 patients, paper CRFs will be digitalized and copies will be saved on at least 2 external disks. For consequent data analysis, we deem single data entry appropriate in this monocentric study.

All EIT and echocardiographic files will be copied from the specific devices on a monthly basis with copies again saved on at least 2 external disks. Final data storage will be in locked cabinets with restricted access.

Paper CRFs will be stored in a secure place at least 10 years after study close-out, whereas original patient files are digitalized after hospital discharge and will be retained indefinitely as all digitalized study data are.

### Confidentiality {27}

After trial enrolment, every patient receives a pseudonym consisting of letters and serial numbers. From then onwards, personal as well as study-related data will only be collected under this pseudonym in the patient’s individual paper CRF. The list of personal patient data linked to the specific pseudonym will be locked in a separate cabinet with access only by the principal investigator.

Echocardiographic data will be anonymized before data analysis and EIT data will be saved under the corresponding pseudonym (see additional file 1, page 9). For phone surveys after 1 and 6 months, the assessor will receive a list with the patient names including date of birth for a correct identification. However, obtained data of these surveys will again be collected in the corresponding pseudonymized CRF (see Additional file 1, page 14).

In general, all study-related information will be stored securely at the study site and any personal patient information will be either locked in cabinets with limited access or will be only accessible via specific user names and passwords in digital databases.

### Plans for collection, laboratory evaluation, and storage of biological specimens for genetic or molecular analysis in this trial/future use {33}

Aliquots of plasma probes will be labeled with the corresponding pseudonym/time point of collection and stored at -80 °C in the laboratories of the Department for Anesthesiology, BG University Hospital Bergmannsheil, until the final publication of the main and ancillary studies. Eventually, not all of these aliquots will be utilized for evaluation. At this time, no further analysis of this plasma biobank is planned. If future ideas for evaluation might evolve which are not yet specified in the current version of the study protocol, a new positive votum of the local REC as well as additional participant approval must be obtained before analysis (see {26b}).

## Statistical methods

### Statistical methods for primary and secondary outcomes {20a}

The intervention group (perioperative individualized FCV) will be compared with best clinical practice PCV as the control group for all primary analyses. The primary study endpoint will be assessed with a linear regression model using the intervention group as an independent variable and the EuroSCORE II as an additional covariable. For all continuous secondary endpoints, similar linear regression models will be fitted. All binary secondary outcomes will be calculated using a logistic regression model as well as SIRS-free days using a Poisson regression model with the same independent variables. For the analyses of multiple secondary outcomes, the Bonferroni method for appropriate adjustment of the overall significance will be utilized.

For all confirmative statistical analyses, 2-sided *p*-values with a significance level of 0.05 will be utilized. *P*-values will be reported to four decimal places with *p*-values less than 0.001 reported as *p* < 0.001. All analyses will be conducted with the statistical software R (www.r-project.org, version ≥ 4.1.0).

### Interim analyses {21b}

Not applicable as no interim analyses is planned for the ongoing study.

### Methods for additional analyses (e.g., subgroup analyses) {20b}

A recent clinical crossover trial has shown the beneficial effects of short-term FCV compared to conventional volume-controlled ventilation with respect to regional ventilation distribution in obese patients with a BMI ≥ 30 kg/m^2^ [7]. Therefore, we plan to conduct a subgroup analysis to compare the effect of individualized FCV or best clinical practice PCV on perioperative lung function, EIT data, and PPCs as well as PEPCs in the setting of perioperative mid-term ventilation of obese patients with a BMI ≥ 30 kg/m^2^ in cardiac surgery. In accordance with the results of Weber et al. [7] we do not only anticipate an enhancement of dorsal ventilation distribution but also of lung oxygenation indices at least after CPB when ventilating with FCV compared to PCV. The analyses on outcome parameters like oxygenation indices, EIT data, PPCs, and PEPCs will be carried out in an explorative approach as only few or no data exist until now. However, in accordance with {20a} for all continuous endpoints, a linear regression model using the intervention group as an independent variable and, for all binary secondary outcomes, logistic regression models will be fitted.

### Methods in analysis to handle protocol non-adherence and any statistical methods to handle missing data {20c}

In this first larger trial of mid-term FCV application in high-risk cardiac surgery procedures, more than one cardiopulmonary bypass time, intraoperative death, and/or cardiopulmonary resuscitation which are exclusively due to major intraoperative surgical complications as well as study protocol deviations with respect to the allocated intervention for more than 5 min (see {11d}) are defined as stop criteria. It is almost impossible that the perioperative ventilatory mode has an impact on the etiology of these stop criteria, especially as lungs are not being ventilated during CPB in the current study protocol. However, major surgical complications leading to the defined stop criteria and the incidence of the criteria itself significantly affect postoperative patient outcomes. Thus, these patients will be excluded from data analysis whereas all other participant data will be analyzed “as randomized” following an intention-to-treat analysis.

Due to the nature of the intervention and the collection of predominantly short-term data, there will be very few missing data regarding secondary outcomes and (nearly) no missing data with respect to the primary endpoint. Since in most cases these will be due to operational disturbances, loss of paper CRFs, and laboratory errors, missing data can be assumed to be completely missing at random. As the omitting of the (few) missing values will not lead to bias this approach will be utilized for this trial.

### Plans to give access to the full protocol, participant-level data and statistical code {31c}

After the publication of main and ancillary study outcomes in peer-reviewed journals the German version of the trial protocol, anonymized participant-level datasets and statistical codes can be obtained from the principal investigator upon personal written request.

## Oversight and monitoring

### Composition of the coordinating center and trial steering committee {5d}

The trial steering committee (TSC) designed the study and prepared and approved the final study protocol version (Version 1.3) as well as the amendments (Version 1.4 and 1.5). The TMC is responsible for patient recruitment, randomization, adherence to the protocol and CRF, correct acquisition of data/biological samples and their processing, for the reporting of severe adverse advents to the local REC, and for organizing TSC meetings and budget administration.

The data managers verify data and maintain trial IT systems including the online-based software REDCap® for randomization.

### Composition of the data monitoring committee, its role and reporting structure {21a}

In this single-center trial with a moderate total sample size and no planned interim analysis, a data monitoring committee was neither required by the TSC nor by the local REC.

### Adverse event reporting and harms {22}

In the current study protocol PPCs and PEPCs as potential intervention harms with a possible causal relationship are collected after patient enrolment and will be assessed as important clinical secondary outcomes (see {12}). Compound outcome and single PPCs/PEPCs analyses will be performed (see {12} and {20a}).

Intra- or postoperative death is defined as a severe adverse event and will be reported to the local REC. Additionally, all severe adverse events defined as study stop criteria and any protocol violations due to operational disturbances (see {11d}) will be reported and published along with the results of the main study.

### Frequency and plans for auditing trial conduct {23}

At least every three months during the patient enrolment period the TSC will meet together with members of the TMC in order to review the trial conduct with respect to protocol adherence and occurrence of stop criteria as well as severe adverse events. During these audits, every reporting and any protocol amendment to the local REC (see {25}) will be planned. Additionally, approved amendments will be made public to members of the TMC in written form.

### Plans for communicating important protocol amendments to relevant parties (e.g., trial participants, ethical committees) {25}

Modifications to the study protocol, which has been primarily approved by the local REC with respect to the primary and secondary outcomes, participant eligibility and exclusion criteria, sample size, application of interventions, and any other substantial changes to the protocol, will require an official amendment.

The amendment will be prepared and approved by the TSC and communicated to the TMC. After the reporting to the REC and the approval, modifications will be implemented and made public in the primary trial registry which for this study is the German Clinical Trials Register. This registry also allows for tracking the emergence of any amendments to the protocol.

Personnel changes or redistribution of responsibilities in the TMC with no effect on the study conduct are defined as minor administrative changes. They only need approval by the TSC which again may notify the REC.

### Dissemination plans {31a}

#### Primary outcome paper

After enrolment of the last study participant, the close-out of FLOWVENTIN HEARTSURG will be at the telephone interview 6 months post-surgery of this patient. From then onwards, we estimate 3 to 4 months for finalizing a manuscript about the primary and main secondary study outcome results for submission to an appropriate medical journal. The final manuscript version and the selected journal need the approval of the TSC and all co-authors before submission.

#### Publication of ancillary studies

After the acceptance of the main study paper, the results of ancillary studies are allowed to be published in peer-reviewed journals. Again, final manuscript versions and the selection of an appropriate journal need the assent of the TSC and co-authors.

#### Abstracts and presentations

Every doctoral candidate of the trial is supposed to submit one abstract with preliminary or final study results to an international or national congress before final data publication. Abstract contents need to be communicated to co-authors and the TSC and approved before being submitted.

If participants are willing to be informed about study results and consent to saving their personal email addresses, they will receive a copy of the publication(s) in case of open access.

## Discussion

During on-pump cardiac surgery procedures, lungs do not only need to withstand mid-term invasive ventilation in deep general anesthesia, surgical manipulations, and recurrent phases of apnea with consecutive atelectasis formation and atelectrauma. Lungs also receive a further hit by partial ischemia and reperfusion through the use of CPB, resulting in LIRI. These various injuries and their combination induce a pulmonary and systemic hyperinflammatory state [8, 37] which to a large extent accounts for the common postoperative pulmonary dysfunctions [10]. Clinical studies in the setting of cardiac surgery could demonstrate a reduction of key inflammatory biomarkers when lung protective ventilation strategies with respect to an open-lung approach [22] and combined with low tidal volumes [21] were applied perioperatively. However, the recent multicenter study PROVECS could not show a clinical benefit towards PPCs prevention when comparing a perioperative, generalized open-lung concept with common practice ventilation in cardiac anesthesia [9].

The trial scope of FLOWVENTIN HEARTSURG is to evaluate the new ventilatory mode of FCV in a single center RCT of a medium sample size in a perioperative approach. We did not exclude any preexisting patient comorbidities per se, except for situations with an initially modified (hyper-) inflammatory environment like in acute myocardial infarction and preexisting pneumonia as well as (suspicion of) endocarditis or intake of any preoperative immunosuppressive medication. The same open approach was chosen for the surgery: All types of single and combined surgical procedures with the exception of heart transplantation and assist device implantations are allowed as on-pump interventions. The reasoning behind this was to have a really thorough sample of cardiac surgical patients and procedures as the basis for our study. However, statistical analysis will adequately account for preoperative comorbidities and the severity of surgery as the EuroSCORE II will function as an additional covariable that specifically weighs patient-, cardiac-, and operation-related factors in the analysis of primary and secondary endpoints.

The comparator of FCV is PCV with a personalized PEEP and a DP mobilizing a lung-protective V_t_ of 6–8 ml/kg PBW. This ventilatory approach is in accordance with current national and international guidelines [38, 39] and large preceding and ongoing multicenter trials regarding perioperative individualization of PEEP [12, 20, 40]. For the titration of a C_dyn_-based best PEEP in the study protocol, an incremental PEEP trial is favored above a decremental PEEP trial as being less invasive due to no extensive airway recruitment with potentially detrimental effects at least in patients with ARDS [41]. PEEP is also titrated in the experimental group of FCV. The rationale behind a personalized PEEP is to exceed the lower inflection point of the static/quasi-static inspiratory lung pressure–volume curve in order to reduce the cyclic de-recruitment/atelectrauma [42, 43].

The theoretical lung protection considerations in FLOWVENTIN HEARTSURG differ from those in any other previous clinical trial: FCV application is not only individualized with respect to an open-lung approach regarding a patient-specific PEEP but also a personalized driving pressure (DP). The C_dyn_-based best DP is obtained during an incremental DP trial which will only be continued to the next DP level if V_t_ has increased disproportionately compared to the C_dyn_ on the previous DP level. This method shall prevent exceeding the upper inflection point of the inspiratory pressure–volume curve where lung compliance begins to decrease at high peak airway pressures resulting in at least regional lung tissue overinflation. With this approach the present theorem of lung-protective tidal volumes is deliberately disregarded as other determinants of mechanical power in the etiology of VILI are prioritized: the respiratory rate and airway flows [2, 43]. Through customization the DP in FCV, respiratory rate and airway flows can be reduced to a minimum still ensuring normocapnia, thereby reducing mechanical power and also dissipated energy of invasive ventilation [3, 4, 26]. Thus far, this approach has only been evaluated in a pre-clinical model of mid-term invasive ventilation resulting in better lung function and aeration compared to conventional PCV with protective tidal volumes and a fixed PEEP [4]. To our best knowledge, one other clinical trial also assesses the intraoperative application of FCV in the setting of cardiac surgery independently from our group [44]. However, the study focus is completely different to the perioperative approach of our study as the stated endpoints are only applicable to a short postoperative timeframe. On top of that the total sample size is rather low.

In order to adhere to the group-specific ventilatory protocols participants are accompanied by members of the TMC from in- to extubation. Preoperative PEEP and DP trials, the intraoperative period, and the postoperative individualization of ventilation are even always conducted by one anesthesiologist in both groups. This guarantees a maximum of uniform consistency in intervention application. From extubation onwards, assessors are blinded to interventions and any further medical therapy is at the discretion of the physician in charge. This approach also includes the handling of interventions classified as PPCs and PEPCs and therefore important secondary study outcomes. For example, in the case of postoperative hypoxemia, we did not define specific cut-off values or indications for non-invasive ventilation and this therapy will be decided upon the discretion of the medical doctor in charge. This might be a drawback in the study protocol as the variability in medical decisions might be increased.

Additionally, the study protocol includes the evaluation of perioperative lung aeration by electrical impedance tomography (EIT) embedded in an independent ancillary study of FLOWVENTIN HEARTSURG. EIT is a relatively new technique that allows for non-invasive point-of-care, two-dimensional visualization of air movements within the thoracic cavity without exposure to radiation. This allows for repetitive real-time assessments but implies a far lower resolution compared to current computed tomography analysis. EIT measurements can be performed on spontaneously breathing and mechanically ventilated patients which is the case in the study protocol. Half of the perioperative EIT recordings are under spontaneous breathing conditions (preoperative baseline and postoperatively after extubation as well as 24 h after CPB end) whereas the other half under invasive ventilation (after individualization of group-specific ventilatory settings preoperatively, after postoperative ICU admission and on pressure support ventilation during respiratory weaning). A similar perioperative approach of EIT measurements as in this study protocol has been recently published as a substudy of the PROVECS trial [33]. However, the authors focused on the results of only two EIT-derived parameters (dorsal fraction of ventilation and regional lung compliance) at four different time points up to the second postoperative day. The current study protocol comprises 5 numerical EIT-derived indices at 6 perioperative time points. With this approach, we hope not only to shed light on the effect of the interventions but also the course of respiratory “milestones” on the way through a cardiac surgery procedure and the subsequent intensive care period.

Regarding the echocardiographic evaluation of right ventricular function and pulmonary circulation, the respective substudy represents a nearly complete set of systolic right ventricular function parameters as described in the most recent guidelines for cardiac chamber quantification by echocardiography in adults by the American Society of Echocardiography and the European Association of Cardiovascular Imaging in 2015 [31].

With the study protocol of the main and ancillary trials, we hope to enable a holistic overview of the implications on lung function, aeration, PPCs, and PEPCs, as well as on cardiovascular side effects when ventilating on-pump cardiac surgery patients perioperatively with the new ventilation mode of FCV. The study FLOWVENTIN HEARTSURG is the largest clinical RCT on FCV thus far. The results of the application of individualized FCV compared to the best clinical PCV will be of high importance for our general understanding of perioperative invasive ventilation and the concomitant VILI.

## Trial status

The current version of the study protocol is version 1.5, the first patient was enrolled on 10 August 2020 and the trial FLOWVENTIN HEARTSURG is currently recruiting. We plan to stop recruitment in November 2022.

## Supplementary Information

**Additional file 1.** Case Report Form FLOWVENTIN HEARTSURG.

## Data Availability

Investigators responsible for analysis of the main study will have full access to the corresponding final dataset as will investigators of the ancillary studies to their specific dataset. Upon request to the TSC clarifying the intention, investigators may be allowed to access other datasets. In order to maintain confidentiality datasets will only include pseudonymized study data.

## Abbreviations

BIPAP: Biphasic positive airway pressure
C_dyn_: Dynamic compliance
CPB: Cardiopulmonary bypass
CPAP: Continuous positive airway pressure
CRF: Case report form
DP: Driving pressure
EIT: Electrical impedance tomography
ELISA: Enzyme-linked immunosorbent assay
FCV: Flow-controlled ventilation
FiO_2_: Fraction of inspired oxygen
ICU: Intensive care unit
IL-8: Interleukin 8
LIRI: Lung ischemia/reperfusion injury
PBW: Predicted body weight
PCV: Pressure-controlled ventilation
PEEP: Positive end-expiratory pressure
PEPCs: Postoperative extra-pulmonary complications
PPCs: Postoperative pulmonary complications
RCT: Randomized controlled trial
REC: Research Ethics Committee
SIRS: Systemic inflammatory response syndrome
TEE: Transesophageal echocardiography
TMC: Trial Management Committee
TSC: Trial Steering Committee
TTE: Transthoracic echocardiography
VCV: Volume-controlled ventilation
VILI: Ventilator-induced lung injury
V_t_: Tidal volume
