# Supplementary material for: Individualized flow-controlled versus conventional pressure-controlled ventilation in on-pump heart surgery (FLOWVENTIN HEARTSURG): study protocol for a randomized controlled trial
Source: Trials. 2023 Mar 16;24:195. doi: 10.1186/s13063-023-07201-7 (PMC10018968; doi:10.1186/s13063-023-07201-7)
Supplement: Supplementary file 1 — Additional file 1. Case Report Form FLOWVENTIN HEARTSURG. [file 13063_2023_7201_MOESM1_ESM.pdf]

Case Report Form

FLOWVENTIN HEARTSURG

Version 1.4

Pseudonym: FVHS - _____ Date: ________ EuroSCORE: ____

Body weight: _____kg **PBW:** □♀: 45,5 + 0,91 * (height – 152,4) = _____kg

Body height: _____cm □♂: 50,0 + 0,91 * (height – 152,4) = _____kg

KOF: _____ m^2

EIT-Belt-Size: _____ Periop. V_T (6-8 ml/kg): _______ml

Tick Boxes

CPB-End:_________

□ $T_{Baseline}$ (before intubation)
- □ art.: BGA, 1 gr. EDTA, 1 Li-Hep., 1 Serum, 1 Diff.-BB, 1 kl. EDTA (BNP)
- □ TTE
- □ EIT

□ T_{-2} (after intubation)
- □ EIT
- □ art. BGA
- □ zv-BGA, 1 gr. EDTA, 1 Li-Hep., 1 Diff.-BB (before skin incision)
- □ TEE 1
- □ ventilation + catecholamines

□ T_{-1} (15 min after thoracotomy)
- □ art. BGA
- □ zv-BGA, 1 gr. EDTA, 1 Li-Hep., 1 Diff.-BB

□ ventilation + catecholamines (1x/h)

CPB

□ catecholamines (1x/h)
□ CPB + clamping duration

□ T_0
- □ catecholamines for CPB-weaning
- □ art. BGA
- □ zv-BGA, 1 gr. EDTA, 1 Li-Hep., 1 Diff.-BB

□ ventilation + catecholamines

□ T_1
- □ art. BGA
- □ zv-BGA, 1 gr. EDTA, 1 Li-Hep., 1 Diff.-BB
- □ TEE 2

□ ventilation + catecholamines

$T_{Baseline\ ITS}$
- □ art. BGA
- □ zv-BGA, EDTA (BNP)
- □ EIT

ICU in process

□ ventilation + catecholamines (1x/h)
□ art. BGA + EIT: before extubation on CPAP + after extubation

□ T_6
- □ art. BGA
- □ zv-BGA, 1 gr. EDTA, 1 Diff.-BB

□ T_{12}
- □ art. BGA
- □ zv-BGA, 1 gr. EDTA

□ T_{24}
- □ art. BGA
- □ zv-BGA, 1 gr. EDTA, 1 Li-Hep., 1 Diff.-BB, 1 kl. EDTA (BNP)
- □ EIT

POD 1
- □ CK, CK-MB, Trop
- □ ICU-Scores

POD 3
- □ CK, CK-MB, Trop, BNP, DiffBB
- □ ICU-Scores

POD 5
- □ CK, CK-MB, Trop, BNP, DiffBB
- □ ICU-Scores

Hospitalisation in process

□ Horovitz indices POD 1-7
□ PPCs POD 1-7
□ PEPCs POD 1-7
□ length of ICU, IMC + normal ward stays
□ infections with AB-treatment during hospitalisation

Telefonsurveys

□ 1 month post surgery
□ 6 months post surgery

Miscellaneous:

TTE / TEE

Parameter	Sub-Parameter	TTE	TEE 1	TEE 2
**RIMP**	**TCOT** Tricuspid Valve Closure to Opening Time	ms	ms	ms
	**RVET** RV Ejection time	ms	ms	ms
Pathologisch > 0,55				
**S`**		cm / s	cm / s	cm / s
	**Echo-Mode**			
Pathologisch < 10 cm/s	**Schnittebene**			
**TAPSE**		cm	cm	cm
	**Echo-Mode**			
Pathologisch ≤ 1,7	**Schnittebene**			
**RVOT FS**	**RVOT enddiastolisch**	cm	cm	cm
	**RVOT endsystolisch**	cm	cm	cm
		%	%	%
	**Echo-Mode**			
O.p.B. > 32 %	**Schnittebene**			
**FAC**	**EDA** End diastolic area of RV	cm^2	cm^2	cm^2
	**ESA** End systolic area of RV	cm^2	cm^2	cm^2
Pathologisch < 35 %		%	%	%
**Speckle Tracking**		File number:	File number:	File number:
	**Echo-Mode**			
	**Schnittebene**			

TTE / TEE

Parameter	Sub-Parameter	TTE			
RA Druck	VCI-Durchmesser	□ ≤ 2,1 cm	□ ≤ 2,1 cm	□ > 2,1 cm	□ > 2,1 cm
	Kollaps in % bei Sniff	□ > 50 %	□ < 50 %	□ > 50 %	□ < 50 %
	RAP	□ 3 mmHg	□ 8 mmHg		□ 15 mmHg

Parameter	Sub-Parameter	TTE		TEE 1		TEE 2	
ZVD / RA Druck		mmHg		mmHg		mmHg	
PAP_{syst}	Peak TR velocity [m/s]	Insp:	Exsp:	Insp:	Exsp:	Insp:	Exsp:
	$PAP_{syst} = 4 * V^2 + ZVD$	Insp:	Exsp:	Insp:	Exsp:	Insp:	Exsp:
PAP_{diast}	End-diastolic PR velocity	Insp:	Exsp:	Insp:	Exsp:	Insp:	Exsp:
	$PAP_{diast} = 4 * V^2 + ZVD$	Insp:	Exsp:	Insp:	Exsp:	Insp:	Exsp:
PAP_{mean}	Early PR velocity	Insp:	Exsp:	Insp:	Exsp:	Insp:	Exsp:
	$PAP_{mean} = 4 * V^2 + ZVD$	Insp:	Exsp:	Insp:	Exsp:	Insp:	Exsp:
	Mean gradient TR aus VTI TR-Jet	Insp:	Exsp:	Insp:	Exsp:	Insp:	Exsp:
	PAP_{mean} = mittl. Gradient + ZVD	Insp:	Exsp:	Insp:	Exsp:	Insp:	Exsp:
	Pulm. acceleration time	Insp:	Exsp:	Insp:	Exsp:	Insp:	Exsp:
	PAP_{mean} lt. Mahan-Formel	Insp:	Exsp:	Insp:	Exsp:	Insp:	Exsp:
	$PAP_{mean} = 1/3\ PAP_{syst} + 2/3\ PAP_{diast}$	Insp:	Exsp:	Insp:	Exsp:	Insp:	Exsp:
PVR	Peak TR velocity [m/s]	Insp:	Exsp:	Insp:	Exsp:	Insp:	Exsp:
	VTI RVOT [cm]	Insp:	Exsp:	Insp:	Exsp:	Insp:	Exsp:
	PVR= (TRVmax/RVOT VTI)*10+0,16	Insp:	Exsp:	Insp:	Exsp:	Insp:	Exsp:
	PVR in $dyn*sec*cm^{-5}$	Insp:	Exsp:	Insp:	Exsp:	Insp:	Exsp:

Mahan-Formel:
AT ≥ 120 ms: PA ~ 79 – (0,45 x AT)
AT < 120 ms: PA ~ 90 – (0,62 x AT)

FVHS-001

Ventilation

Intubation: _______

Begin CPAP: _______

Begin Weaning: _______

Extubation : _______

Time on mechanical ventilation:_____min

Weaning time: _____min

Para-meter	T -2		T -1				T 0	T 1	T 2	T 3	T 4	T 5	T 6	T 7	T 8	T 9	T 10	T 11	T 12	T 13	T 14	T 15	T 16	T 17	T 18	T 19	T 20	T 21	T 22	T 23	T 24
**Time**																															
**Mode**																															
**LPM / Application**																															
V_T																															
**AF**																															
**AMV**																															
**FLOW**																															
**PEEP**																															
**PIP** Pinsp																															
**DP / ASB** PIP - PEEP																															
P_{mean}																															
C_{dyn}																															
**R**																															
**I : E**																															

Catecholamines

CPB Begin: ________

CPB End : ________

Clamping: ________

De-Clamping:_______

CPB time : ________min

Clamping time: _______min

Para-meter	Boli	T -2		T -1				Boli	T 0	T 1	T 2	T 3	T 4	T 5	T 6	T 7	T 8	T 9	T 10	T 11	T 12	T 13	T 14	T 15	T 16	T 17	T 18	T 19	T 20	T 21	T 22	T 23	T 24
Time																																
NOR																																
EP																																
TP																																
DOB																																
MR																																
SUPRA																																
SIMDAX																																
Ilomed.																																
Akrinor																																
ZVD																																
PPV																																
PAP_{Sys} PAP_{Dia} PAP_{Mean}																																

Art. BGA

Parameter	$T_{Baseline}$	T_{-2}	T_{-1}		T_0	T_1	$T_{Beginn\ ITS}$		T_6		CPAP (ASB ≤ 6)	1. BGA spontan	T_{12}	T_{24}
Time														
F_iO_2											0,4 !!			
pCO_2														
pO_2														
Lactate														
tHb														
sO_2														
BE_{ecf}														
$HCO_3^-{}_{std}$														

zv-BGA

Parameter		T_{-2}	T_{-1}		T_0	T_1	$T_{Beginn\ ITS}$		T_6				T_{12}	T_{24}
Time														
pO_2														
tHb														
sO_2														

Data derived from BGAs + PAC

Parameter	Formula	$T_{Baseline}$	T_{-2}	T_{-1}	T_0	T_1	$T_{Beginn ITS}$	T_6	CPAP	Extu-bation	T_{12}	T_{24}
Air pressure	hPa / mmHg											
Horovitz	PaO_2 / FiO_2											
$AaDO_2$	$PAO_2 - PaO_2$											
CaO_2	$Hb*1,34*SaO_2 + PaO_2*0,0031$											
DO_2	$HZV* CaO_2 * 10$											
CvO_2	$Hb*1,34*SvO_2+PvO_2*0,0031$											
VO_2	$HZV*(CaO_2 - CvO_2) * 10$											
O_2-ER	VO_2 / DO_2											
PAO_2	$(Patm - PH_2O) * FiO_2 - (PaCO_2/RQ)$											
CcO_2	$Hb*1,34*SaO_2 + PAO_2*0,0031$											
Qs/Qt	$\frac{CcO_2 - CaO_2}{CcO_2 - CvO_2}$											

Central lab

Parameter	$T_{Baseline}$	T_{-2}	T_{-1}	T_0	T_1	$T_{Beginn ITS}$	T_6	T_{24}	T_{3d}	T_{5d}
Date/Time										
CK	U/l					U/l		U/l	U/l	U/l
CK-MB	U/l					U/l		U/l	U/l	U/l
Troponin	pg/ml					pg/ml		pg/ml	pg/ml	pg/ml
BNP	pg/ml					pg/ml		pg/ml	pg/ml	pg/ml
Leukos	/nl	/nl	/nl	/nl	/nl		/nl	/nl		

Differential blood count

Parameter	$T_{Baseline}$	T_{-2}	T_{-1}	T_0	T_1	T_6	T_{24}	POD 3	POD 5
Date / Time									
Segmentkernige	/nl	/nl	/nl	/nl	/nl	/nl	/nl	/nl	/nl
Eosinophile	/nl	/nl	/nl	/nl	/nl	/nl	/nl	/nl	/nl
Basophile	/nl	/nl	/nl	/nl	/nl	/nl	/nl	/nl	/nl
Lymphos	/nl	/nl	/nl	/nl	/nl	/nl	/nl	/nl	/nl
Monos	/nl	/nl	/nl	/nl	/nl	/nl	/nl	/nl	/nl
Erythroblasten	/nl	/nl	/nl	/nl	/nl	/nl	/nl	/nl	/nl
Segmentkernige	%	%	%	%	%	%	%	%	%
Eosinophile	%	%	%	%	%	%	%	%	%
Basophile	%	%	%	%	%	%	%	%	%
Lymphos	%	%	%	%	%	%	%	%	%
Monos	%	%	%	%	%	%	%	%	%
Erythroblasten	%	%	%	%	%	%	%	%	%

C-reactive protein

	Preop.	Begin ICU	POD 1	POD 2	POD 3	POD 4	POD 5	POD 6	POD 7
Date / Time									
CRP	mg/dl	mg/dl	mg/dl	mg/dl	mg/dl	mg/dl	mg/dl	mg/dl	mg/dl

EIT I

Filename : FVHS-001_Calibration

**Event**	PEEP:<br>DP:	PEEP:<br>DP:	PEEP:<br>DP:	PEEP:<br>DP:	PEEP:<br>DP:	PEEP:<br>DP:	PEEP:<br>DP:	PEEP:<br>DP:	PEEP:<br>DP:	PEEP:<br>DP:	PEEP:<br>DP:	PEEP:<br>DP:	PEEP:<br>DP:	PEEP:<br>DP:	PEEP:<br>DP:	PEEP:<br>DP:
**V_T** [/min]																
**Tidalrate** [ml]																
**Compliance**																
**Resistance**																

Filename : FVHS-001_Study

	$T_{Baseline}$		T_{-2}		$T_{Begin\ ICU}$		T_{CPAP}		$T_{Extubation}$		T_{POD1}	
**Date/Time**												
**Event**	**Base**		**T-2**		**ICU**		**CPAP**		**Ext**		**POD1**	
**ROI setting**	Layers	Quadrants	Layers	Quadrants	Layers	Quadrants	Layers	Quadrants	Layers	Quadrants	Layers	Quadrants
**ROI 1**												
**ROI 2**												
**ROI 3**												
**ROI 4**												
**PEEP**			mbar		mbar		mbar					
**P_{Max}**			mbar		mbar		mbar					
**V_T**			ml		ml		ml					
**Tidalrate**	/ min		/ min		/ min		/ min		/ min		/ min	
**Compliance**												
**Resistance**												

Fluid balances

Parameter	Intraop.	24 pm POD 0	24 pm POD 1	24 pm POD 3	24 pm POD 5
Date					
HLM-balance					
All infusions					
Free infusions					
Transfusions					
Urinary output					
**TOTAL**					

Art. BGA

Parameter	POD 1	POD 2	POD 3	POD 4	POD 5	POD6	POD7
Date / Time (~12 a.m.)							
F_iO_2							
pH							
$PaCO_2$							
PaO_2							
PaO_2 / F_iO_2 (Horovitz)							

Postoperative pulmonary complications (PPCs)

ARDS: Berlin definition 2012

Timing	Within 1 week of a known clinical insult or new or worsening respiratory symptoms
Chest imaging[a]	Bilateral opacities—not fully explained by effusions, lobar/lung collapse, or nodules
Origin of edema	Respiratory failure not fully explained by cardiac failure or fluid overload<br>Need objective assessment (eg, echocardiography) to exclude hydrostatic edema if no risk factor present
Oxygenation[b]	
Mild	200 mm Hg < PaO_2/FIO_2 ≤ 300 mm Hg with PEEP or CPAP ≥5 cm H_2O[c]
Moderate	100 mm Hg < PaO_2/FIO_2 ≤ 200 mm Hg with PEEP ≥5 cm H_2O
Severe	PaO_2/FIO_2 ≤ 100 mm Hg with PEEP ≥5 cm H_2O

Suspected pneumonia

- New pulmonary infiltrate on chest x-ray

AND ≥ 2 following items
- temperature > 38.5 / < 35.5°C
- Leucocytes > 12 / nl or < 4 / nl
- purulent secretions and AB

Parameter	POD 0	POD 1	POD 2	POD 3	POD 4	POD 5	POD6	POD7
**Date**								
**Invasive ventilation**	□ Y □ N	□ Y □ N	□ Y □ N	□ Y □ N	□ Y □ N	□ Y □ N	□ Y □ N	□ Y □ N
**Non-invasive ventilation**	□ Y □ N	□ Y □ N	□ Y □ N	□ Y □ N	□ Y □ N	□ Y □ N	□ Y □ N	□ Y □ N
**Respiratory acidosis**<br>(pH ≤ 7.3 and $PaCO_2$ > 45 mmHg)	□ Y □ N	□ Y □ N	□ Y □ N	□ Y □ N	□ Y □ N	□ Y □ N	□ Y □ N	□ Y □ N
**Reintubation**	□ Y □ N	□ Y □ N	□ Y □ N	□ Y □ N	□ Y □ N	□ Y □ N	□ Y □ N	□ Y □ N
**Readmission to ICU**	□ Y □ N	□ Y □ N	□ Y □ N	□ Y □ N	□ Y □ N	□ Y □ N	□ Y □ N	□ Y □ N
**ARDS** (Berlin definition)<br>**- mild ARDS**<br>**- moderate ARDS**<br>**- severe ARDS**	□ Y □ N<br>□ Y □ N<br>□ Y □ N	□ Y □ N<br>□ Y □ N<br>□ Y □ N	□ Y □ N<br>□ Y □ N<br>□ Y □ N	□ Y □ N<br>□ Y □ N<br>□ Y □ N	□ Y □ N<br>□ Y □ N<br>□ Y □ N	□ Y □ N<br>□ Y □ N<br>□ Y □ N	□ Y □ N<br>□ Y □ N<br>□ Y □ N	□ Y □ N<br>□ Y □ N<br>□ Y □ N
**VV - ECMO**	□ Y □ N	□ Y □ N	□ Y □ N	□ Y □ N	□ Y □ N	□ Y □ N	□ Y □ N	□ Y □ N
**Suspected pneumonia**	□ Y □ N	□ Y □ N	□ Y □ N	□ Y □ N	□ Y □ N	□ Y □ N	□ Y □ N	□ Y □ N
**Confirmed pneumonia**	□ Y □ N	□ Y □ N	□ Y □ N	□ Y □ N	□ Y □ N	□ Y □ N	□ Y □ N	□ Y □ N
**Bronchoschopy**<br>**Reason:**	□ Y □ N	□ Y □ N	□ Y □ N	□ Y □ N	□ Y □ N	□ Y □ N	□ Y □ N	□ Y □ N
**Pleural drainage due to pleural effusions**	□ Y □ N	□ Y □ N	□ Y □ N	□ Y □ N	□ Y □ N	□ Y □ N	□ Y □ N	□ Y □ N
**Pleural drainage due to pneumothorax**	□ Y □ N	□ Y □ N	□ Y □ N	□ Y □ N	□ Y □ N	□ Y □ N	□ Y □ N	□ Y □ N

Postoperative extra-pulmonary complications (PEPCs)

SIRS: ≥ 2 of following criteria
- Temperature > 38 or < 36°C
- Heart rate > 90 bpm
- Respiratory rate > 22 or $PaCO_2$ < 32 mmHg
- Leucocytes > 12 / nl or < 4 / nl

Sepsis:
- acute Δ in SOFA-Score ≥ 2

Septic shock:
- need for vasopressors for MAP ≥ 65
AND
- lactate level > 2 mmol / L

Singer et al. The third international consensus definition for sepsis and septic shock. JAMA 2016; 315: 801-10.

Parameter	POD 0	POD 1	POD 2	POD 3	POD 4	POD 5	POD6	POD7
COPY of ECG	□ Y	□ Y	□ preoperative ECG					
Date								
SIRS	□ Y □ N	□ Y □ N	□ Y □ N	□ Y □ N	□ Y □ N	□ Y □ N	□ Y □ N	□ Y □ N
Sepsis	□ Y □ N	□ Y □ N	□ Y □ N	□ Y □ N	□ Y □ N	□ Y □ N	□ Y □ N	□ Y □ N
Septic shock	□ Y □ N	□ Y □ N	□ Y □ N	□ Y □ N	□ Y □ N	□ Y □ N	□ Y □ N	□ Y □ N
Coronary intervention	□ Y □ N	□ Y □ N	□ Y □ N	□ Y □ N	□ Y □ N	□ Y □ N	□ Y □ N	□ Y □ N
Postop. wound infection with AB	□ Y □ N	□ Y □ N	□ Y □ N	□ Y □ N	□ Y □ N	□ Y □ N	□ Y □ N	□ Y □ N
Other infectious site with AB	□ Y □ N	□ Y □ N	□ Y □ N	□ Y □ N	□ Y □ N	□ Y □ N	□ Y □ N	□ Y □ N
Pericardial tamponade with intervention	□ Y □ N	□ Y □ N	□ Y □ N	□ Y □ N	□ Y □ N	□ Y □ N	□ Y □ N	□ Y □ N
Bleeding with intervention	□ Y □ N	□ Y □ N	□ Y □ N	□ Y □ N	□ Y □ N	□ Y □ N	□ Y □ N	□ Y □ N
IABP	□ Y □ N	□ Y □ N	□ Y □ N	□ Y □ N	□ Y □ N	□ Y □ N	□ Y □ N	□ Y □ N
Impella	□ Y □ N	□ Y □ N	□ Y □ N	□ Y □ N	□ Y □ N	□ Y □ N	□ Y □ N	□ Y □ N
VA-ECMO	□ Y □ N	□ Y □ N	□ Y □ N	□ Y □ N	□ Y □ N	□ Y □ N	□ Y □ N	□ Y □ N
ICU-readmission (non-pulmonary)	□ Y □ N	□ Y □ N	□ Y □ N	□ Y □ N	□ Y □ N	□ Y □ N	□ Y □ N	□ Y □ N

Stay

	POD 0	POD 1	POD 3	POD 5
Date / Time				
Ward				

KDIGO-Score

Preop. Crea: _______

Parameter	POD 0	POD 1	POD 3	POD 5
Crea				
Urine				
KDIGO				

AKI-Stadium	S-Kreatinin	Urin-Ausscheidung
1	1,5- bis 1,9-facher Anstieg innerhalb von sieben Tagen oder Anstieg ≥ 0,3 mg/dl innerhalb von 48 Stunden	< 0,5 ml/kg/h über mehr als sechs Stunden
2	2,0- bis 2,9-facher Kreatininanstieg	< 0,5 ml/kg/h über mehr als zwölf Stunden
3	≥ 3-facher Kreatininanstieg oder Serum-Kreatinin ≥ 4 mg/dl mit einem akuten Anstieg ≥ 0,5 mg/dl	< 0,3 ml/kg/h über mehr als 24 Stunden oder fehlende Urinausscheidung (Anurie) für ≥ zwölf Stunden

CAM-ICU

	Merkmal I	Merkmal II	Merkmal III	Merkmal IV	Delir	Ø Delir
POD 1	☐	☐	RASS:	☐	☐	☐
POD 3	☐	☐	RASS:	☐	☐	☐
POD 5	☐	☐	RASS:	☐	☐	☐

Point of care

	ICU	IMC	Normal ward	PODs until discharge	AHB	Hospital transfer
Duration	d	d	d	d	☐	☐

Telephone surveys

	1 Monat postop.	6 Monate postop
**Datum**		
**Stationäre Aufnahme**	☐	☐
**In AHB**	☐	☐
**Stationär geblieben**	☐	☐
**Grund für Aufnahme**		
**Diagnose**		
**Freitext**		
